# The Role of Gut Microbiota—Gut—Brain Axis in Perioperative Neurocognitive Dysfunction

**DOI:** 10.3389/fphar.2022.879745

**Published:** 2022-06-14

**Authors:** Jian Lu, Wenlong Hou, Sunan Gao, Ye Zhang, Youming Zong

**Affiliations:** ^1^ Department of Anesthesiology, The Second Hospital of Jiaxing, The Second Affiliated Hospital of Jiaxing University, Jiaxing, China; ^2^ Department of Anesthesiology, Bengbu Medical College, Bengbu, China

**Keywords:** gut microbiota, gut-brain axis, perioperative neurocognitive dysfunction, postoperative cognitive dysfunction, cognition

## Abstract

With the aging of the world population and advances in medical and health technology, more and more elderly patients are undergoing anesthesia and surgery, and perioperative neurocognitive dysfunction (PND) is receiving increasing attention. The latest definition of PND, published simultaneously in November 2018 in 6 leading journals in the field of anesthesiology, clarifies that PND includes preoperatively cognitive impairment, postoperative delirium, delayed neurocognitive recovery, and postoperative cognitive dysfunction and meets the diagnostic criteria for neurocognitive impairment in the Diagnostic and Statistical Manual of Mental Disorders -fifth edition (DSM-5). The time frame for PND includes preoperatively and within 12 months postoperatively. Recent studies have shown that gut microbiota regulates central nervous function and behavior through the gut microbiota - gut - brain axis, but the role of the axis in the pathogenesis of PND remains unclear. Therefore, this article reviews the mechanism of the role of gut microbiota-gut-brain axis in PND, so as to help explore reasonable early treatment strategies.

## Introduction

**FIGURE 1:**
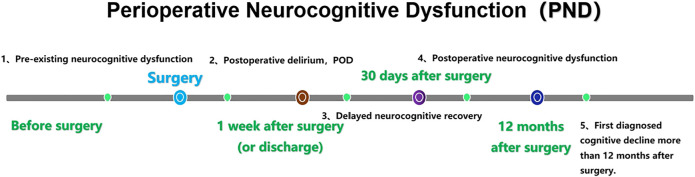
Nomenclature of PND. PND is divided into five categories by time period, including preoperative neurocognitive dysfunction (preoperative, measurable, objective impairment of cognitive function), POD (occurred within 1 week after surgery or before discharge and met the DSM-5 diagnostic criteria for delirium), delayed neurocognitive recovery (cognitive decline within 0–30 days after surgery), postoperative neurocognitive disfunction (pNCD or POCD, mild and severe cognitive decline existed from 30 days to 12 months after surgery) and first diagnosed cognitive decline more than 12 months after surgery. Gut microbiota dysbiosis and intestinal barrier dysfunction.

PND refers to alterations in cognitive function before and/or after surgery, including preoperatively diagnosed cognitive decline, postoperative delirium (POD), delayed neurocognitive recovery, and postoperative cognitive dysfunction (POCD) (Evered et al., 2018a). (Figure 1). The incidence of PND rangs from 41 to 75% at 7 days postoperatively to 18–45% at 3 months postoperatively (Austin et al., 2019). PND tends to occur in elderly patients and is mainly characterized by diminished attention, memory, and verbal thinking skills. The risk factors for PND are multifaceted and may be related to age, infection, preexisting cognitive disorders, surgery duration, anesthetic management, tissue damage, psychological stress and genetic susceptibility (Subramaniyan and Terrando, 2019; Eckenhoff et al., 2020). It has been shown that PND is mainly associated with neuroinflammation, cholinergic dysfunction, oxidative stress, abnormal accumulation of β-amyloid and impaired neurosynaptic function (Pepeu and Giovannini, 2010; Lin et al., 2020). PND is very harmful as it can lead to prolonged hospital stays, increased hospital costs, high social burden and increased mortality. Currently, there are many prevention and treatment strategies for PND, including drug prevention (Su et al., 2011; Tian et al., 2015; Lee et al., 2017; Zhang et al., 2018), antibiotic anti-inflammatory (Liang et al., 2018), control of fasting time and carbohydrate load (Batchelor et al., 2019), physical activity and social participation (Lai et al., 2021), anesthesia management, such as multi-mode analgesia including preemptive analgesia, dexmedetomidine, and epidural analgesia (Lei et al., 2020; Fan et al., 2021), and blood pressure management (Maheshwari et al., 2020) and so on. However, these strategies have not achieved ideal clinical effects. Therefore, it is particularly important to explore the pathogenesis and effective prevention and treatment strategies of PND.

In recent years, gut microbiota has become an important topic in biological research. Signals from the gut can regulate the function of specific brain regions, and patients with central nervous system (CNS) diseases are often accompanied by gastrointestinal symptoms such as constipation and diarrhea, so the concept of gut microbiota - gut - brain axis emerged. Germ-free mice can show impaired learning and memory (Diaz Heijtz et al., 2011; Clarke et al., 2013), and recent studies have shown that changes in gut microbiota are associated with abnormal cognitive behavior. The abnormal gut microbiota caused by surgical anesthesia was age-dependent, which manifested by a significant decrease in the abundance and diversity of the microbiota with increasing age (Liufu et al., 2020). The reduction of beneficial bacteria (*lactic acid bacteria, bifidobacterium*) increases the risk of postoperative cognitive impairment, while surgical anesthesia exacerbates gut microbiota dysbiosis and shifts gut microbiota to a more toxic phenotype (Guyton and Alverdy, 2017). Many perioperative factors can affect the gut microbiota, including the operation itself, antibiotics, opioids or acid-inducing drugs (Krezalek and Alverdy, 2016). Studies have found a strong link between abnormal gut microbiota composition and the onset of autism, depression, schizophrenia and Alzheimer’s disease (Mangiola et al., 2016; Lv et al., 2017; Yang et al., 2017a; Yang et al., 2017b; Zhan et al., 2018). There is growing evidence that gut microbes communicate with the central nervous system and can influence brain function and behavior through neural, endocrine, and immune pathways (Lynch and Hsiao, 2019). Thus, it would be beneficial for the prevention and treatment of CNS diseases to regulate gut microbiota composition and improving its physiological functions.

Intestinal barrier dysfunction is considered to be an important cause of gut-brain axis dysfunction. Normal gut microbiota is equivalent to a protective biological barrier for the intestine, while gut microbiota dysbiosis can not only produce neurotoxic factors, but also affect intestinal mucosal permeability, resulting in “leaky gut”, which can cause a large number of inflammatory and neurotoxic factors to enter the CNS (Osadchiy et al., 2019). The phenomenon of “leaky gut” with increased intestinal permeability and gut microbiota dysbiosis occurs after general anesthesia surgery and is closely related to postoperative cognitive impairment.

### Gut Microbiota Dysbiosis and Neuroimmune Dysfunction

Central nervous inflammatory response is the main pathologic process of PND (Nathan, 2019; Yang et al., 2019; Yang et al., 2020a). Tissue damage and oxidative stress induced by surgery and anesthesia can induce the release of local or systemic pro-inflammatory cytokines and the activation of corresponding inflammatory signaling pathways, resulting in systemic inflammation (Huber-Lang et al., 2018). Pro-inflammatory cytokines can utilize specific receptors and transporters on the surface of endothelial cells of the blood-brain barrier (BBB) and directly cross the BBB, triggering neuroinflammation and ultimately leading to POCD (Abrahamov et al., 2017; Yang S. et al., 2017; Yang et al., 2020a). Microglia are resident immune cells of the CNS, responsible for immune monitoring and detection of their designated brain regions (Kettenmann et al., 2011). As systemic inflammation progresses, microglia develop an activated morphological phenotype and release increased pro-inflammatory factors such as interferon-γ (IFN-γ), interleukin-1β (IL-1β), tumor necrosis factor-α (TNF-α), and reactive oxygen species (ROS) (Liu et al., 2020). Microglia release proinflammatory factors that contribute to subsequent astrocyte activation and further promote neuroinflammation (Liddelow et al., 2017; Liu et al., 2020). In addition, cytokines entering the brain can activate the hypothalamic-pituitary-adrenal (HPA) axis to release cortisol and act on downstream pathway of the glucocorticoid receptor in the hippocampus, triggering depressive-like behaviors (Luo et al., 2018). Activation of complement system is another necessary inflammatory response activated by the damage associated molecular patterns triggered by surgery. For example, deposition of c-reactive protein activates and modulates the classical complement pathway, leading to inflammatory dysregulation. Use of drugs that block the complement cascades, such as C3 receptor blockers, improves neuroinflammation and memory function in PND (Xiong et al., 2018), also suggesting the role of neuroinflammation in PND.

**FIGURE 2:**
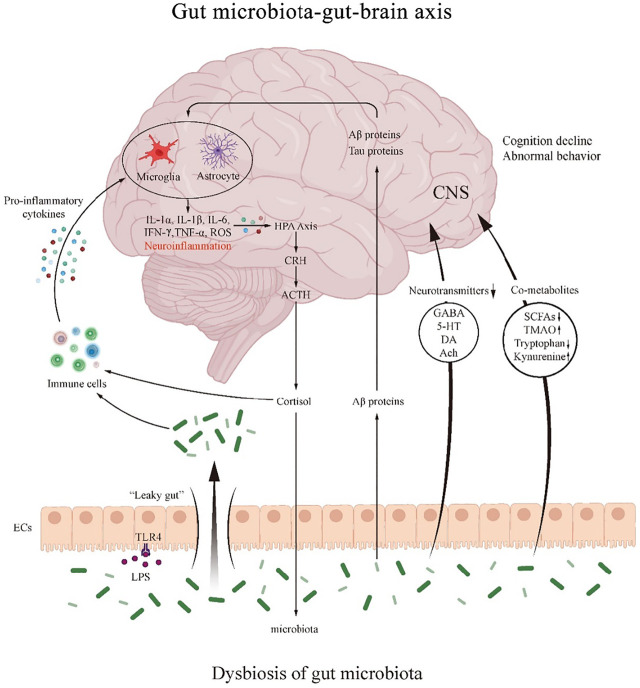
The mechanism of the role of gut microbiota-gut-brain axis in PND. The gut microbiota regulates the interactions between the gut and brain through neural, endocrine and immune pathways. Dysbiosis of the gut microbiota regulates immune activity and produces pro-inflammatory cytokines that can either promote the formation of neuroinflammation and further activate the HPA axis.

Gut microbiota dysbiosis aggravates postoperative systemic inflammatory response. Intestinal mucosal immune barrier is an important component of the intestinal barrier, mainly composed of abundant lymphocytes and macrophages, and is the largest immune cells reservoir of the body. The normal gut microbiota maintains a good balance with the host mucosal immune system. A systematic retrospective study shows that patients with or without digestive surgery experienced significant changes in their gut microbiota, characterized by an increased proportion of gram-negative bacteria (Lederer et al., 2017). Gram-negative bacterial cell wall lipopolysaccharide (LPS) activates Toll-like receptors 4 (TLR4) on the surface of intestinal epithelial cells and mediates enhanced intestinal permeability, the activation of TLR in plasma and brain cells by LPS can induce the release of pro-inflammatory cytokines and lead to memory deficits (Yang et al., 2020b). At the same time, dendritic cells directly capture the antigens of intestinal dysregulated bacteria, or Intestinal Microfold Cells phagocytose antigens and then deliver them to dendritic cells and other antigen-presenting cells, which affect the development and differentiation of CD4^+^ and CD8^+^ T cells, resulting in the imbalance of intestinal immune homeostasis (Ekmekciu et al., 2017). The damaged intestinal barrier can then promote intestinal bacteria and intestinal toxic metabolites to enter the blood circulation. Pro-inflammatory factors can reduce the expression of tight junction proteins such as occludin and claudin-5 at mRNA and protein levels, destroy the integrity of BBB, and can enter the brain to activate adaptive immune cells, resulting in brain immune dyshomeostasis (Ni et al., 2018). In animal studies, oral *lactobacillus* reduced the BBB permeability, and consequently protected the postoperative cognitive functions of the aged and gut dysbiosis mice (Wen et al., 2020). And after oral prebiotic Bimuno^®^ (galactooligosaccharide (B-GOS) mixture), the number of probiotics such as *Lactobacillus and Bifidobacterium* in intestine of PND mice increased, the level of inflammatory factors in hippocampus of brain decreased, and the cognitive function improved (Yang et al., 2018). A meta-analysis suggested that probiotics enhanced cognitive performance in patients with Alzheimer’s disease (AD) or mild cognitive impairment (MCI) possibly by reducing levels of inflammatory and oxidative biomarkers (Den et al., 2020). It was found that probiotics VSL#3 treatment could upregulate the expression of microRNA-146a (miR-146a) and block the BTG2/Bax (B-cell translocation gene 2/Bcl-2-associated X protein) axis in POCD mice, thus inhibiting neuronal apoptosis and reducing oxidative stress (Mao et al., 2021). In addition, miR-146a overexpression alleviated hippocampal dependent learning and memory impairment and hippocampal inflammation in POCD mice (Chen et al., 2019). MicroRNA-146a protects neurons by altering microglial phenotypes, reducing pro-inflammatory cytokines and enhancing phagocytosis, thereby ameliorating cognitive deficits in AD mice (Liang et al., 2021). Therefore, postoperative gut microbiota dysbiosis may promote the development of PND by aggravating peripheral and central inflammatory responses. (Figure 2).

### Gut Microbiota Dysbiosis and Neurotransmitter Products

The occurrence of PND is closely related to the concentration of neurotransmitters in brain. Neurotransmitters such as acetylcholine (Ach), 5-hydroxytryptamine (5-HT), dopamine (DA), gamma amino butyric acid (GABA), etc. affect the function of CNS through central cholinergic and dopaminergic nerves, and the decline of learning and memory ability is often accompanied by the changes in neurotransmitter levels in related brain regions. Application of cardiopulmonary bypass (CPB) can reduce the mortality caused by myocardial infarction, heart failure, and fatal arrhythmias, but CPB-related complications are prevalent, such as POCD and gastrointestinal injury. α7nAchR activation markedly reduced intestinal injury, POCD, neuronal apoptosis, proinflammatory factor expression, and number of CD4^+^ IL-17^+^ cells. In contrast, the lack of α7nAChR significantly aggravated the pro-inflammatory response and POCD caused by CPB (Kong et al., 2015; Chen et al., 2018). There is increasing evidence that the pathophysiology of delirium is caused by multiple neurotransmitter system dysfunctions. The concentrations of DA and 5-HT metabolites in cerebrospinal fluid (CSF), hippocampus and basolateral amygdala of POD rats were significantly increased. After treatment with the selective 5-HT1A antagonist WAY-100635, POD symptoms were reversed at some extent in rats (Qiu et al., 2016).

Surgical anesthesia disturbs the gut microbiota, destroys the 5-HT synthesis and metabolic homeostasis involved in intestinal endocrine cells and gut microbiota, and can lead to the fluctuation of the level of 5-HT in the body, thus affecting the mood, behavior and postoperative gastrointestinal peristalsis. In addition, *lactobacillus, Bifidobacterium*, *Streptococcus* and other bacteria in the intestinal tract are involved in the process of GABA synthesis by glutamic acid metabolism, and the GABA synthesized by gut microbiota directly stimulates enterochromaffin cells to secrete 5-HT, affecting the levels of brain-derived nutritional factors and dopamine. Supplementing probiotics, regulating gut microbiota and promoting the stability of neurotransmitter level will be beneficial to postoperative recovery (Yang et al., 2020b; Lv et al., 2021). Fructooligosaccharides have been found to have positive neural effects in AD rats, such as increasing Ach, serotonin, and adrenaline, and reducing damage to the CA1 region of the hippocampus (Chen et al., 2017). Therefore, postoperative gut microbiota dysbiosis may promote the development of PND by affecting the balance of neurotransmitters. (Figure 2).

### Gut Microbiota Dysbiosis and Abnormal Gut Microbiota-Host Co-Metabolites

Gut microbiota can provide the host with biological enzymes and biotransformation pathways that the human body does not possess, and co-metabolize with the host, producing metabolites such as vitamins, fatty acids, bile acids and trimethylamine N-oxide (TMAO) and other metabolites through various metabolic pathways and transferring them to the circulatory system, thus regulating the microenvironment and function of the brain.

Vitamin D (VD) plays a vital role in gut homeostasis (Malaguarnera, 2020). VD administration improved cognitive dysfunction by modulating gut microbiota and increased *Bacteroidetes/Firmicutes* ratio (Hussein et al., 2022). VD supplementation increased the relative abundance of *Bacteroides* (Charoenngam et al., 2020). Vitamin A deficiency exacerbated learning and memory deficits, decreased abundance of *Lactobacillus* and α- and β-diversity. Gut microbiota α diversity decreased, β diversity increased in depressed bipolar disorder patients, mainly for the abundance levels of *Streptococcaceae* and *Bacteroidaceae* increased, while the abundance levels of *Akkermansia Muciniphila* and *Faecalibacterium prausnitzii* decreased. Meanwhile, further analysis showed that the level of serum metabolites changed significantly in depressed bipolar disorder patients, including B-vitamins, kynurenic acid, gamma-aminobutyric acid and short-chain fatty acids. These metabolites may be associated with the abundance of gut microbiota, including *Akkermansia muciniphila, Citrobacter spp, Phascolarctobacterium spp, Yersinia spp, Enterobacter spp* and *Flavobacterium spp* (Li et al., 2022).

Short-chain fatty acids (SCFAs) are essential metabolites of intestinal microbial activity and are trophic factors for the intestinal mucosa and epithelium. They are produced by microbial fermentation of dietary fiber in colon, and mainly include acetate (C_2_), propionate (C_3_) and butyrate (C_4_) (Martin-Gallausiaux et al., 2021). SCFAs induce inflammation and immune responses by activating G protein-coupled receptors (GPR41 and GPR43) on the surface of intestinal epithelial cells and immune cells (Xiong et al., 2004; Kimura et al., 2013). SCFAs activate the sympathetic nervous system by binding to the GPR41 receptor in sympathetic ganglion neurons (Kimura et al., 2011). SCFAs can cross BBB, affect neurotransmission and neurotransmitter production, and induce abnormal behavior (DeCastro et al., 2005; Mitchell et al., 2011). Microbiota can also regulate the metabolism of tryptophan, which plays a key role in the normal operation of the immune system and gut-brain axis (Agus et al., 2018), the three major metabolic pathways for the production of kynurenine, indole and 5-HT from tryptophan are all under the indirect or direct control of microbiota. DA, Ach and GABA can also be produced by gut microbiota (Fung et al., 2017; Sherwin et al., 2018). In addition, LPS, a metabolite of bacteria, can directly affect the CNS by activating TLR4 in microglia, resulting in massive production of inflammatory cytokines in the CNS (Leitner et al., 2019). In mice with AD, fecal microbiota transplantation (FMT) treatment reversed the changes of gut microbiota and the SCFA butyrate, and the symptoms were improved (Sun et al., 2019).

TMAO, a gut microbe-dependent metabolite is implicated in the development of age-related cognitive decline. Increased levels of circulating TMAO may induce the susceptibility to surgery, contributing to aggravating neuroinflammation and decreasing cognitive ability in elderly rats after surgery (Meng et al., 2019). Reduced circulating TMAO levels by 3,3-Dimethyl-1-butanol ameliorated the cognitive decline in APP/PS1 mice, accompanying a decrease in neuroinflammation and the Amyloid β (Aβ)1–42 levels in the hippocampus (Gao et al., 2019). The study found that a decrease in the number of *lactobacillus plantarum* in the gut was associated with cognitive impairment. *Lactobacillus plantarum* enhanced the therapeutic effects of memantine treatment in APP/PS1 mice by remodeling the intestinal microbiota and inhibiting the synthesis of TMAO (Wang et al., 2020).

Gut microbiota metabolites have been proved to have neuroactive or toxic effects. Recent studies have shown significant metabolic abnormalities in patients with postoperative cognitive impairment. Therefore, postoperative gut microbiota dysbiosis may promote the development of PND by causing abnormal gut microbiota-host co-metabolites. (Figure 2).

### Gut Microbiota Dysbiosis and Amyloid-Beta and Tau Protein

Advanced age is an independent risk factor for PND, and neuronal degeneration may occur in elderly patients before surgery, which is manifested by Aβ protein accumulation and protein hyperphosphorylation in the brain. The accumulation of Aβ protein and hyperphosphorylation of Tau protein were increased in neurons of aged mice after surgery and anesthesia (Terrando et al., 2011). In animal studies, isoflurane exposure led to spatial memory impairment with increases of levels of Aβ and phosphorylated Tau protein in the brain (Zuo et al., 2018). In addition, it has been reported that elevated Aβ and Tau protein in CSF after surgery are risk factors for PND (Evered et al., 2018b). The accumulation of Aβ and Tau protein induces neuroinflammation, leading to glial cell activation, pro-inflammatory cytokines release and neuronal damage (Calsolaro and Edison, 2016).

Aβ proteins from the gut microbiota themselves (produced by *E. coli, Bacillus subtilis, Salmonella*, etc.) can enter bloodstream through the damaged intestinal wall. Although the primary structure of enteric-derived Aβ is different from that of cerebral Aβ, its tertiary structure is very similar, suggesting that enteric-derived Aβ may trigger cross-immune responses and trigger overactivation of pro-inflammatory signaling pathways in the brain. The increased proportion of *Escherichia coli* in the intestinal tract after surgery and anesthesia can promote Aβ protein deposition inside and outside the neuronal cells, and can promote the synaptic dysfunction and even lead to cell death by activating the reactive changes of glial cells surrounding the neuronal cells. In addition, deposition of Aβ protein interferes with NMDA receptor mRNA expression in hippocampal neurons and cortex, reducing synaptic plasticity and leading to cognitive impairment (Newcombe et al., 2018). The increase in Aβ protein caused by gut microbiota dysbiosis can be ameliorated by transplantation of normal fecal microbiota. Germ-free mice showed cognitive dysfunction after receiving FMT from human amyloid precursor protein knock-in mice, which indirectly indicated that gut microbiota dysbiosis could promote Aβ protein deposition to cause cognitive impairment (Kundu et al., 2022). Therefore, postoperative gut microbiota dysbiosis may promote the development of PND by causing abnormal accumulation of Aβ and Tau protein. (Figure 2).

### Gut Microbiota Dysbiosis and Anxiety and Depression

Anxiety and depression are the most common psychiatric disorders with increasing numbers of people worldwide suffering from them (Kessler and Bromet, 2013). Anxiety, depression and functional bowel disorders are highly comorbid, suggesting that the gut-brain axis may be involved in the pathological mechanism of these psychosis (Mussell et al., 2008). Patients with anxiety and depression often present with elevated inflammation levels, HPA axis dysfunction, neurotransmitter signaling dysfunction, etc. As previously described, gut microbiota regulates these events, and thus they may have the potential to regulate depression and anxiety disorders (Dinan and Cryan, 2013).

Studies have shown that gut microbiota can regulate anxiety in mice. For example, compared with the SPF control, GF Swiss Webster, National Institutes of Health Swiss, and Naval Medical Research Institute mice showed decreased anxiety-like behavior. Conversely, GF BALB/c and C57BL/6 mice showed increased anxiety-like behavior (Sampson and Mazmanian, 2015). These results suggested that anxiety-like behavior in mice was highly correlated with gut microbiota.

A growing number of studies have shown that people with major depressive disorder (MDD) have altered gut microbiota structure in comparison with healthy controls. Gut microbiota dysbiosis can lead to depression-like behaviors in GF mice. For example, GF mice exhibited depressive-like behaviors and metabolic disturbances after receiving FMT from MDD patients (Kelly et al., 2016; Zheng et al., 2016). Thus gut microbiota dysbiosis is an important factor causing MDD.

Recently, a large amount of evidence has shown the potential anti-anxiety and anti-depression activity of probiotics. Treating mice with *Lactobacillus rhamnosus* JB-1 reduced stress-induced HPA response and anxiety-like behavior (Bravo et al., 2011). Administration of *Lactobacillus helveticus* improved anxiety and depression caused by restraint stress in adult SPF rats (Liang et al., 2015). Moreover, the combination of *Lactobacillus helveticus* R0052 and *Bifidobacterium longum* R0175 showed potential anti-anxiety like activity in rats and promoted psychological properties in healthy people (Messaoudi et al., 2011). *Bifidobacterium longum* 1714 or *Bifidobacterium breve* 1205 reduced stress-related behavior (anxiety or depression) in innately anxious BALB/c mice (Savignac et al., 2014). Prebiotics have also been shown to improve anxiety and depression. Galacto-oligosaccharides (GOS) or the combination of GOS and fructo-oligosaccharide improved anxious and depressant behavior in rodents (Burokas et al., 2017).

Patients with PND often present with a variety of neuropsychiatric symptoms like anxiety and depression, and anxiety and depression will accelerate the decline of their cognitive function (Ismail et al., 2018). We already know that gut microbiota is linked to anxiety and depression, so gut microbiota may promote the development of PND by inducing neuropsychiatric symptoms such as anxiety and depression.

Gut microbiota dysbiosis leads to altered levels of neurotransmitters, such as GABA, 5-HT, DA and Ach. It also causes abnormal gut microbiota-host co-metabolites modulating SCFAs, TMAO and tryptophan/kynurenine pathway metabolites. Gut microbiota and LPS affect intestinal mucosal permeability, resulting in “leaky gut”. Aβ proteins from the gut microbiota themselves can enter bloodstream through the damaged intestinal wall and play a role in neuroinflammation.

CRH: corticotropin releasing hormone; ACTH: adrenocorticotropic hormone; HPA: hypothalamic-pituitary-adrenal; EC: enteroendocrine cell; CNS: central nervous system; GABA: gamma amino butyric acid; 5-HT: 5-hydroxytryptamine; DA: dopamine; Ach: acetylcholine; SCFA: short-chain fatty acids; TMAO: trimethylamino oxide; LPS: lipopolysaccharide; TLR4: toll-like receptor 4.

## Conclusion

In summary, there is a strong link between gut microbiota and PND that depends on the gut-brain axis. Gut microbiota modulates neurological function in the brain through various pathways such as participation in immune regulation and neuroendocrine regulation (Xu et al., 2020), affecting patients’ cognitive function. Mechanisms by which gut microbiota regulate the CNS have been proposed, including the biotransformation of neurotoxicants mediated by gut microbiota, changes in neuroactive products in response to environmental stressors, bidirectional communication within the gut brain axis to alter the integrity of the intestinal barrier and regulation of mucosal immune function (Dempsey et al., 2019). Surgery and anesthesia may disrupt gut microbiota homeostasis in a direct or indirect manner, causing cognitive dysfunction. The research on PND and gut microbiota dysbiosis is getting more and more advanced, but there is a lack of research on gut microbiota alterations and postoperative cognitive changes after surgery and anesthesia, and more basic and clinical research is needed in the future to focus on the role of specific gut microbiome and specific targets of gut microbiota and provide new approaches for the treatment of PND.
